# Impact of the COVID-19 Pandemic on Middle-Aged and Older Patients With Adolescent Idiopathic Scoliosis Who Underwent Spinal Fusion: A Questionnaire-Based Survey

**DOI:** 10.7759/cureus.34370

**Published:** 2023-01-30

**Authors:** Tsutomu Akazawa, Toshiaki Kotani, Tsuyoshi Sakuma, Yasushi Iijima, Yoshiaki Torii, Jun Ueno, Atsuhiro Yoshida, Hisateru Niki, Seiji Ohtori, Shohei Minami

**Affiliations:** 1 Department of Orthopaedic Surgery, St. Marianna University School of Medicine, Kawasaki, JPN; 2 Department of Orthopaedic Surgery, Seirei Sakura Citizen Hospital, Sakura, JPN; 3 Department of Orthopaedic Surgery, Graduate School of Medicine, Chiba University, Chiba, JPN

**Keywords:** long-term, oswestry disability index (odi), srs-22, spinal-fusion, adolescent idiopathic scoliosis (ais), coronavirus disease 2019 (covid-19)

## Abstract

Purpose

To investigate the impact of the COVID-19 pandemic on middle-aged and older patients with adolescent idiopathic scoliosis (AIS) who underwent spinal fusion.

Methods

The subjects were 252 AIS patients who underwent spinal fusion between 1968 and 1988. The surveys were performed before the COVID-19 pandemic (a primary survey in 2014) and during the pandemic (a secondary survey in 2022). The self-administered questionnaires were mailed to the patients. We analyzed 35 patients (33 females and two males) who replied to both surveys.

Results

The pandemic had low impacts on 11 patients (31.4%). Two patients reported refraining from seeing a doctor because they were concerned about going to the clinic or hospital, eight reported that the pandemic impacted their work, and five reported fewer opportunities to go out (based on multiple-choice answers). Twenty-four patients reported that their lives were unaffected by the pandemic. No significant differences were detected between both surveys for Scoliosis Research Society-22 (SRS-22) in any domains (function, pain, self-image, mental, or satisfaction). The Oswestry Disability Index (ODI) questionnaires revealed a significant worsening of the survey during the pandemic compared with the survey before the pandemic. There was no significant difference in the impact of the pandemic between the ODI deterioration group (27.8%) and the ODI stable group (35.3%).

Conclusion

The COVID-19 pandemic had a low impact on 31.4% of middle-aged and older patients with AIS who underwent spinal fusion. The impact of the pandemic did not significantly differ between the groups with ODI deteriorations and the groups with stable ODI. The pandemic had a smaller impact on AIS patients at a minimum of 33 years after surgery.

## Introduction

Several long-term follow-up studies reported that the postoperative health-related quality of life (QOL) of young adult patients with adolescent idiopathic scoliosis (AIS) was good [1-9]. It was also reported that the surgically treated AIS patients had a good QOL in middle-aged and older patients [10]. These studies indicated that long-term postoperative AIS patients were stable in their daily lives.

The World Health Organization declared the novel coronavirus (COVID-19) outbreak a global pandemic in March 2020. Although the COVID-19 pandemic has greatly impacted social life, there are no reports yet on the impact of the COVID-19 pandemic on postoperative AIS patients. The purpose of our study was to investigate the impact of the COVID-19 pandemic on middle-aged and older AIS patients who underwent spinal fusion. The survey was conducted while the pandemic was ongoing. We investigated the impact of the pandemic on these patients.

## Materials and methods

This study protocol was approved by the Institutional Review Board of Seirei Sakura Citizen Hospital (approval number: 2021020). The subjects were 252 patients with AIS who underwent spinal fusion between 1968 and 1988. The mean age at surgery was 14.8 years (range, 10‒19 years), the mean preoperative Cobb angle was 68.3 degrees (range, 32‒130 degrees), and the mean Cobb angle two years after surgery was 39.4 degrees (range, 6‒90 degrees). The surveys were performed before the COVID-19 pandemic (in 2014) and during the pandemic (in 2022). The self-administered questionnaires were mailed to the patients. The patients completed the questionnaires and returned them after giving their consent to participate in the study. In the survey before the pandemic, 48 patients responded and 62 did not respond, 134 had unknown addresses, and eight died. A secondary survey was conducted on the 48 participants who participated in the primary survey. In the survey during the pandemic, 35 patients (35 of 252 patients: 13.9%) responded and 13 did not respond. We analyzed 35 patients (33 females and two males) who replied to both surveys. The mean age of patients at the time of the secondary survey during the pandemic was 57.4 years (range, 47‒67 years), and the mean postoperative follow-up period was 42.7 years (33‒52 years). The mean body height was 153.5 cm (range, 138.0-165.0). The mean body weight was 49.0 kg (range, 31.2-75.0). The mean body mass index (BMI) was 20.8 (range, 14.3‒32.0). The operative procedures used were Harrington instrumentation in 22 cases, Harrington instrumentation with wiring in six cases, multiple hook instrumentation (Chiba Spinal System, Tanakaikakikai, Tokyo, Japan) [11] in three cases, Zielke instrumentation in three cases, and Dwyer instrumentation in one case. 

We assessed the impact of COVID-19 on daily life, work, illness consultation, and treatment. In the survey during the pandemic, occupational status was assessed, as well as the impact of the COVID-19 pandemic on life. The questionnaires utilized included Scoliosis Research Society-22 (SRS-22) patient questionnaire [12,13] and Oswestry Disability Index (ODI) [14,15]. The SRS-22 and ODI questionnaires were self-administered in the surveys before and during the pandemic. 

For statistical analyses, SPSS version 26.0 (IBM Corp., Armonk, NY, USA) was used. Variables were expressed as the mean ± standard deviation. Statistical analyses included a paired t-test or a Fisher’s exact test with chi-square. Significant differences were defined as p<0.05.

## Results

Impact of the COVID-19 pandemic on life and work

Seventeen patients were unemployed (including housewives), five were employed part-time, seven had work requiring light physical activity, six had work requiring moderate physical activity (such as standing for prolonged periods), and no patient had work requiring heavy physical activity.

**Figure 1:**
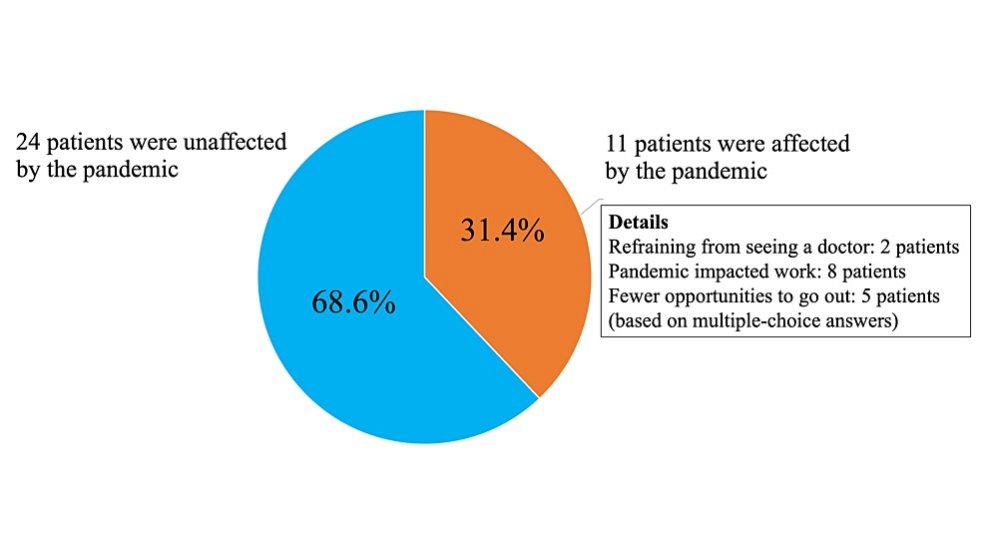
Impact of the novel coronavirus (COVID-19) pandemic on life and work Twenty-four patients (68.6%) reported that their lives were unaffected by the COVID-19 pandemic. The COVID-19 pandemic had some impact on 11 patients (31.4%).

The COVID-19 pandemic had some impact on 11 patients (31.4%). Two patients reported refraining from seeing a doctor because they were concerned about going to the clinic or hospital, eight reported that the pandemic impacted their work, and five reported fewer opportunities to go out (based on multiple-choice answers). Twenty-four patients reported that their lives were unaffected by the COVID-19 pandemic (Figure 1).

Impact of the COVID-19 pandemic on health-related QOL

**Figure 2:**
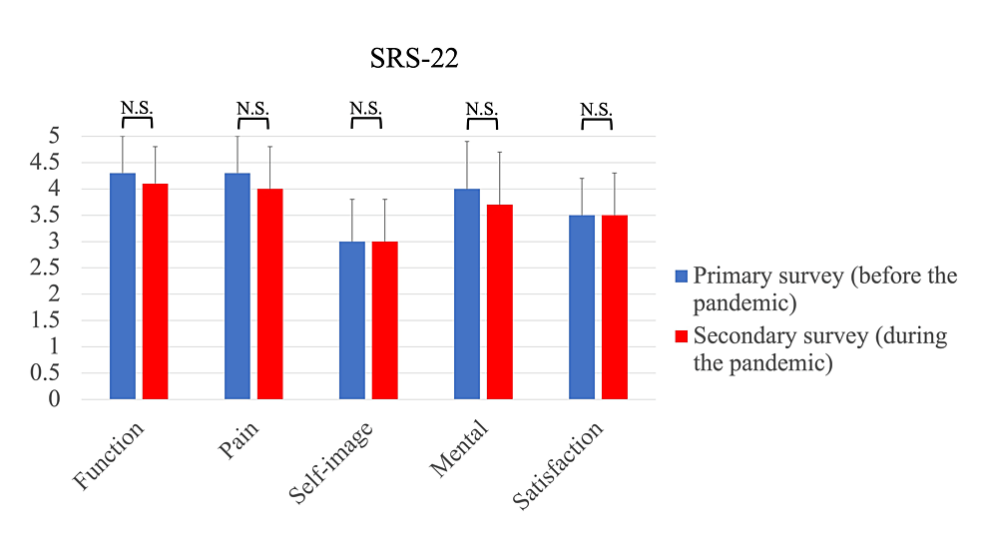
Scoliosis Research Society-22 (SRS-22) patient questionnaire before and during the pandemic The SRS-22 questionnaires revealed that no significant differences were detected between both surveys for any domain.

For the SRS-22 questionnaires, no significant differences were detected between both surveys for function domain (primary survey: 4.3 ± 0.7, secondary survey: 4.1 ± 0.7, p=0.075), pain domain (primary survey: 4.3 ±0.7, secondary survey: 4.0 ±0.8, p=0.073), self-image domain (primary survey: 3.0 ± 0.8, secondary survey: 3.0 ± 0.8, p=0.780), mental domain (primary survey: 4.0 ± 0.9, secondary survey: 3.7 ± 1.0, p=0.057), or satisfaction domain (primary survey: 3.5 ± 0.7, secondary survey: 3.5 ± 0.8, p=0.571) (Figure 2).

**Figure 3:**
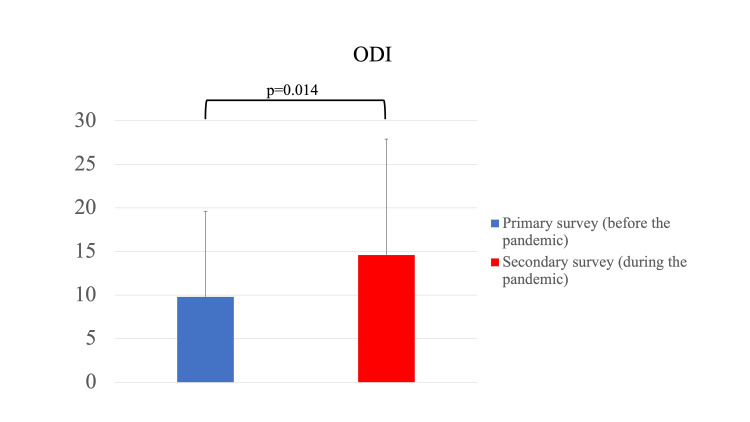
Oswestry Disability Index (ODI) before and during the pandemic The ODI questionnaires revealed a significant worsening of the secondary survey compared with the primary survey.

The ODI questionnaires revealed a significant worsening of the secondary survey compared with the primary survey (primary survey: 9.8 ± 9.8, secondary survey: 14.6 ± 13.3, p=0.014) (Figure 3). The ODI worsened for 18 patients. 

**Figure 4:**
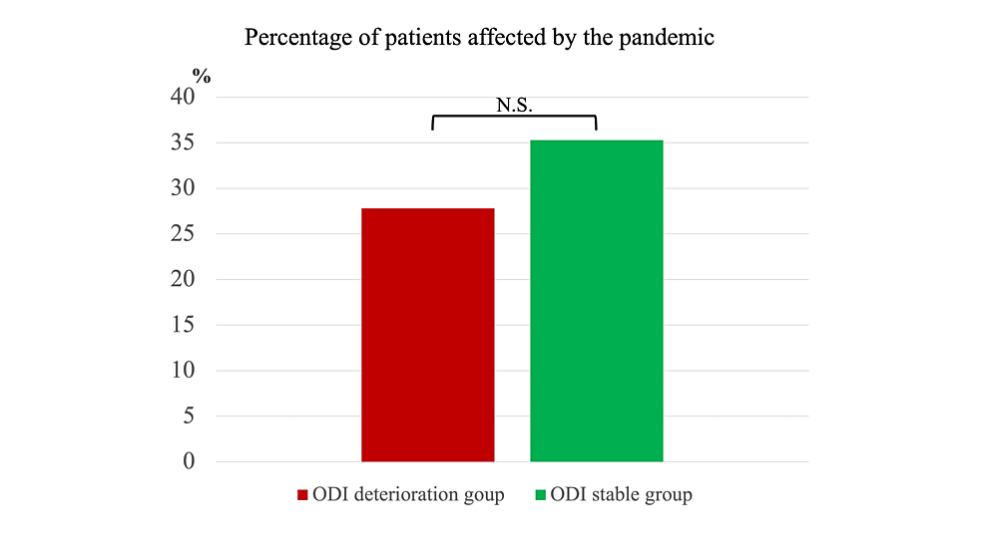
The impact of the pandemic on the Oswestry Disability Index (ODI) deterioration group and the ODI stable group There was no significant difference in the impact of the pandemic between the ODI deterioration group and the ODI stable group.

Eighteen patients with a deterioration of ODI (ODI deterioration group) were compared with 17 patients who had a stable or rising ODI (ODI stable group). There was no significant difference in the impact of the pandemic between the ODI deterioration group and the ODI stable group, as evidenced by five patients (27.8%) versus six patients (35.3%), respectively, being impacted; p=0.454 (Figure 4).

## Discussion

The COVID-19 pandemic had some impact on 31.4% of middle-aged and older patients with AIS at least 33 years after surgery. No significant differences were detected between the survey before and during the pandemic for SRS-22 any domains. The ODI questionnaires revealed a significant worsening of the survey during the pandemic compared with the survey before the pandemic. However, there was no significant difference in the impact of the pandemic between the group with deterioration of ODI and the group with stable ODI. This is the first report on how the COVID-19 pandemic affected long-term patients who had undergone AIS surgery. 

Patients with spinal disorders have been shown to be negatively impacted by the COVID-19 pandemic and the guidance provided required people to reduce their social interactions [16]. It was also reported that 39% of elderly patients with spinal disease had reduced their activities during the pandemic [17]. In our study, 31.4% of the participants claimed the pandemic had affected them in some manner. A survey of postoperative patients with adult spinal deformity reported that confinement during the pandemic had no effect on SRS-22, although a negative effect in relation to ODI was detected [18]. Our study results indicated no correlation between the impact of the COVID-19 pandemic and ODI deterioration.

There are few reports on the impact of COVID-19 on scoliosis patients. According to reports, the initial wave of the COVID-19 pandemic considerably raised the abandonment rates for brace treatments in AIS. Additionally, curve progression and surgical indication were significantly increased in individuals whose brace therapy was stopped [19]. To the best of our knowledge, there have been no reports examining the effects of the COVID-19 pandemic on long-term patients after AIS surgery. According to this study, the pandemic had a smaller impact on AIS patients at least 33 years after surgery. The impact of COVID-19 on these patients was not considered serious.

There were several limitations in this study. Given that this was a lengthy and involved survey that was done twice over an eight-year period, the participation rate was low. Since preoperative and postoperative radiographs were no longer archived, detailed imaging studies could no longer be carried out. Because digital image data is becoming more common, we plan to address the issue of long-term storage. No studies have looked at the minimal clinically meaningful difference (MCID) for long-term changes in SRS-22 or ODI after surgery for AIS, despite reports of MCID values for SRS-22 and ODI for various illnesses and therapies. If the changes in the health-related QOL in our group of patients who experienced a significant decline in their ODI were clinically significant, more study is required to ascertain this point.

## Conclusions

The COVID-19 pandemic had a low impact on 31.4% of middle-aged and older patients with AIS who underwent spinal fusion. There were no significant differences in the SRS-22 domains between the survey conducted before and during the pandemic. According to the ODI surveys, the survey findings during the pandemic significantly worsened from the survey results before the pandemic. However, the impact of the pandemic did not significantly differ between the groups with ODI deteriorations and the groups with stable ODI. The pandemic had a smaller impact on AIS patients at least 33 years after surgery. The next study will focus on whether the worsened ODI returns to the baseline after the pandemic. Future studies using questionnaires would be expected to clarify this point.
